# Genetic Dissection of Behavioural and Autonomic Effects of Δ^9^-Tetrahydrocannabinol in Mice

**DOI:** 10.1371/journal.pbio.0050269

**Published:** 2007-10-09

**Authors:** Krisztina Monory, Heike Blaudzun, Federico Massa, Nadine Kaiser, Thomas Lemberger, Günther Schütz, Carsten T Wotjak, Beat Lutz, Giovanni Marsicano

**Affiliations:** 1 Department of Physiological Chemistry, Johannes Gutenberg University Mainz, Mainz, Germany; 2 Max Planck Institute of Psychiatry, Munich, Germany; 3 Department of Molecular Biology of the Cell I, German Cancer Research Center, Heidelberg, Germany; 4 U862 Institute F. Magendie, University Bordeaux 2, INSERM, Avenir Group 4, Bordeaux, France; University of Texas Southwestern Medical Center, United States of America

## Abstract

Marijuana and its main psychotropic ingredient Δ^9^-tetrahydrocannabinol (THC) exert a plethora of psychoactive effects through the activation of the neuronal cannabinoid receptor type 1 (CB1), which is expressed by different neuronal subpopulations in the central nervous system. The exact neuroanatomical substrates underlying each effect of THC are, however, not known. We tested locomotor, hypothermic, analgesic, and cataleptic effects of THC in conditional knockout mouse lines, which lack the expression of CB1 in different neuronal subpopulations, including principal brain neurons, GABAergic neurons (those that release γ aminobutyric acid), cortical glutamatergic neurons, and neurons expressing the dopamine receptor D1, respectively. Surprisingly, mice lacking CB1 in GABAergic neurons responded to THC similarly as wild-type littermates did, whereas deletion of the receptor in all principal neurons abolished or strongly reduced the behavioural and autonomic responses to the drug. Moreover, locomotor and hypothermic effects of THC depend on cortical glutamatergic neurons, whereas the deletion of CB1 from the majority of striatal neurons and a subpopulation of cortical glutamatergic neurons blocked the cataleptic effect of the drug. These data show that several important pharmacological actions of THC do not depend on functional expression of CB1 on GABAergic interneurons, but on other neuronal populations, and pave the way to a refined interpretation of the pharmacological effects of cannabinoids on neuronal functions.

## Author Summary

Marijuana and its main psychoactive component, THC, exert a plethora of behavioural and autonomic effects on humans and animals. Some of these effects are the cause of the widespread illicit use of marijuana, while others might be involved in the potential therapeutic use of this drug for the treatment of several neuronal disorders. The great majority of these effects of THC are mediated by cannabinoid receptor type 1 (CB1), which is abundantly expressed in the central nervous system. The exact anatomical and neuronal substrates of each action are, however, not clearly known at the moment. We addressed this issue by using an advanced genetic approach. Control and conditional mutant mice, lacking CB1 expression in defined neuronal subpopulations but not in others, were treated with THC, and typical effects of the drug on motor behaviour, pain, and thermal sensation were scored. Our results show that different neuronal subpopulations mediate different effects of THC and could lead to a refined interpretation of the pharmacological actions of cannabinoids. Moreover, these data might provide the rationale for the development of drugs capable of selectively activating CB1 in specific neuronal subpopulations, thereby better exploiting cannabinoids' potential therapeutic properties.

## Abstract 3

Advanced genetics techniques reveal that the effects of cannabinoids on motor behavior and thermal and pain sensation are mediated by distinct populations of glutamatergic and dopaminergic neurons, not GABAergic neurons as previously thought.

## Introduction

Cannabinoids are a class of pharmacological compounds that comprise derivatives of the plant Cannabis sativa (marijuana) and represent one of the oldest known sources of psychotropic drugs [1,2]. Δ^9^-tetrahydrocannabinol (THC) is the prototypical plant-derived psychoactive cannabinoid and the main cause of the psychotropic effects of marijuana, which is the most widespread illicit drug in the world. Administration of cannabinoids to animals and humans induces a complex pattern of behavioural effects, which can be analyzed in laboratory settings [3]. In particular, in mice, cannabinoids produce a specific array of effects in the same dose range and within the same time frame. These effects, consisting of hypolocomotion, hypothermia, antinociception, and catalepsy (impaired ability to initiate movements), represent the so-called “tetrad model” of cannabimimetic activity [3–5]. Despite the fact that the “tetrad” of effects does not exhaustively represent the myriad cannabinoid behavioural and autonomic actions, it is one of the best available measures of cannabimimetic activity of drugs and has been extensively used to identify and classify cannabinoid compounds [3,4]. The psychoactive effects of cannabinoids are mediated by the cannabinoid receptor type 1 (CB1) and, in particular, the tetrad effects of THC are abolished in mutant mice lacking the expression of CB1 [6,7] and are blocked by CB1 antagonists [5,8,9]. Moreover, psychotropic effects of marijuana were shown to be attenuated by blockade of CB1 receptors in humans, confirming the central importance of these receptors in the pharmacology of psychotropic cannabinoids [10].

CB1 is a seven-transmembrane G protein-coupled receptor expressed at very high levels in the central nervous system and at lower levels in peripheral tissues [11,12]. Together with CB2 cannabinoid receptors, CB1 is the molecular target of specific endogenous lipid signalling molecules, the endocannabinoids [13–15]. Cannabinoid receptors, endocannabinoids and the enzymatic machinery for endocannabinoid synthesis and degradation constitute the endocannabinoid system, which is involved in several physiological and pathophysiological processes such as, among many others, retrograde signalling at neuronal synapses [16,17], memory processing [15,17], pain perception [15,18,19], regulation of locomotion [15,18], and inflammation [15,20,21]. In the brain, the expression of CB1 correlates with the psychotropic effects of cannabinoids [11,22]. However, the neuronal mechanisms and the neuronal circuitries responsible for these effects have not yet been clarified. In the brain, where THC and other cannabinoids exert most of their behavioural effects, CB1 receptors are expressed at different levels in different neuronal subpopulations. In particular, CB1 protein and mRNA are present at very high levels in cortical GABAergic interneurons (those that release γ aminobutyric acid [GABA]), where they mediate cannabinoid-dependent inhibition of GABA release [17,23,24]. However, CB1 receptors are also expressed in other neuronal subpopulations, including, among others, glutamatergic cortical principal neurons [24–30]. Given the extraordinary high expression of CB1 on GABAergic interneurons, the modulation of the activity of these neurons is generally believed to mediate most of the effects of exogenously administered and endogenously released cannabinoids [23]. However, due to the lack of suitable experimental tools, this concept has not yet been investigated in vivo.

With the advent of conditional mutagenesis techniques, which are aimed also at obtaining specific deletion of genes in particular cell types [31,32], it is now possible to address directly the involvement of different neuronal populations in the pharmacological effects of cannabinoids. We used recently generated conditional mutant mice for the CB1 receptor, bearing a deletion of the *CB1* gene in principal neurons (defined as projecting neurons as opposed to interneurons, independently of their neurochemical characteristics, normally expressing Ca^2+^/calmodulin-dependent kinase IIα [CaMKIIα] [33,34]), in cortical glutamatergic neurons, and in GABAergic neurons [26,27]. Furthermore, we produced a new mouse mutant line where the Cre-mediated deletion of the *CB1* gene is driven by the regulatory sequences of the D1 dopamine receptor. All of these conditional *CB1* mouse mutants were tested in the “tetrad” battery of THC effects. The results indicate that the typical pharmacological effects of cannabinoids rely on complex anatomical substrates and that, at odds with previous concepts, GABAergic interneurons appear not to be involved in these effects.

## Results

### Locomotor, Hypothermic, Analgesic, and Cataleptic Effects of THC Depend on CB1 Receptors

**Figure 1:**
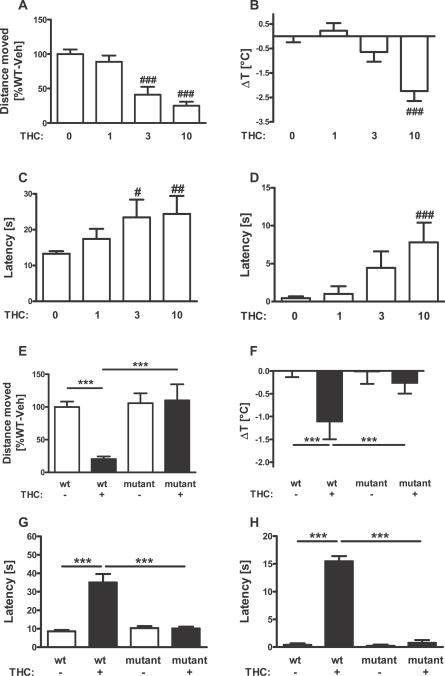
Hypolocomotor, Hypothermic, Analgesic, and Cataleptic Effects of THC Depend on CB1 Receptors (A–D) Dose-response of THC effects in wild-type mice. Effects of vehicle (*n* = 16), 1 mg/kg (*n* = 9), 3 mg/kg (*n* = 9), and 10 mg/kg THC (*n* = 10), respectively, on the “tetrad” battery of tests consisting of (A) locomotor, (B) hypothermic, (C) analgesic, and (D) cataleptic effects. (E–H) THC effects depend on CB1 receptor. Wild-type *CB1* ^+/+^ and littermate *CB1* ^−/−^ mice (i.e., null *CB1* mutants) were tested for (E) locomotor, (F) hypothermic, (C) analgesic, and (D) cataleptic effects of 10 mg/kg THC. Note the absence of any effect of the drug in *CB1* ^−/−^. #, ##, ###; p < 0.05, p < 0.01, p < 0.001, respectively, as compared to vehicle-treated animals (two-way ANOVA, followed by Dunnet's post-hoc test). ***, p < 0.001 (two-way ANOVA, followed by Newman-Keuls post-hoc test).

The “tetrad” effects of THC were shown to depend on the expression of CB1 receptors, because these effects are abolished in CB1-null mutant mice and are blocked by CB1 antagonists [5,7–9]. However, discrepancies in the phenotype of *CB1*-null mutant lines were observed in different experimental setups, likely due to differences of test conditions and genetic background of the mice [6,7]. To verify that the “tetrad” effects of THC fully depend on CB1 expression under our experimental conditions and in our strain of *CB1*-null mutants, we first performed a dose-response (0, 1, 3, and 10 mg THC/kg mouse body weight [mg/kg]) study of these effects of THC in mice and then used the dose of 10 mg/kg on *CB1* ^−/−^ mice and their littermates *CB1* ^+/+^ [35]. The results clearly showed that THC dose-dependently exerts the “tetrad” effects in our experimental conditions (Figure 1A–1D) and that the deletion of CB1 does not alter per se the observed basal conditions of vehicle-treated animals, but fully abolished the effects of THC (Figure 1E–1H; interaction genotype × treatment, F_1,34_ > 9.6, *p* < 0.004).

### Generation of Conditional Mutant Mice Lacking CB1 in Dopamine Receptor D1-Expressing Neurons

**Figure 2:**
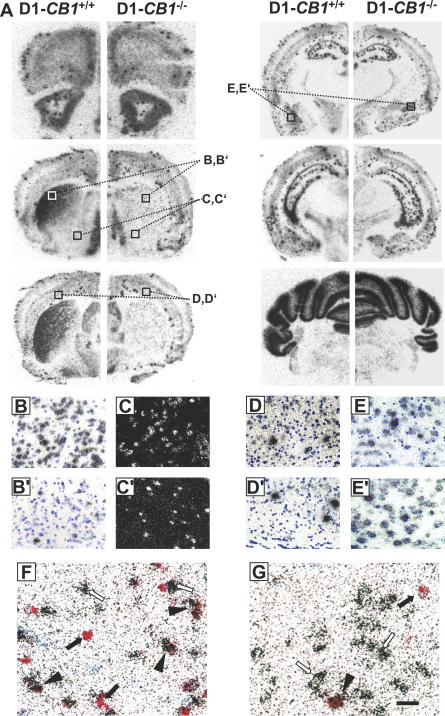
CB1 Receptor mRNA Distribution in D1-*CB1* ^−/−^ Mice (A) Bright-field photomicrographs showing CB1 mRNA expression in coronal brain sections of wild-type D1-*CB1* ^+/+^ (left panels) and D1-*CB1* ^−/−^ mice (right panels). (B–E′) Micrograph showing high magnifications of the details indicated in (A). (B and B′) Detail (corresponding to the areas depicted in (A)) of CB1 expression in the dorsolateral caudate putamen of D1-*CB1* ^+/+^ (B) and D1-*CB1* ^−/−^ (B′). Note the strong decrease in CB1-expressing cells in mutant mice. (C and C′) Dark-field micrographs showing detail (corresponding to the areas depicted in (A)) of CB1 mRNA expression in the ventral striatum of D1-*CB1* ^+/+^ (C) and D1-*CB1* ^−/−^ (C′). (D andD′) Bright-field micrographs showing the expression of CB1 mRNA in layer VI of neocortex (corresponding to the areas depicted in (A)). Note the similar number of cells expressing CB1 at high levels (intense concentration of silver grains) between genotypes and the decreased amount of cells expressing CB1 at low levels in D1-*CB1* ^−/−^ mice (see text for quantifications and details). (E and E′) No evident difference in the levels of CB1 expression were observed in any other cortical regions, as exemplified in the basolateral amygdala. (F and G) Micrographs showing CB1 mRNA (red staining) together with dopamine receptor D2 (silver grains) in the dorsal striatum of D1-*CB1* ^+/+^ (F) and D1-*CB1* ^−/−^ mice (G). Note the reduction of CB1-expressing neurons and the presence of both dopamine receptor D2-positive and D2-negative CB1-containing cells in D1-*CB1* ^−/−^ mice (see text for quantifications and details). Filled arrows, single CB1-expressing neurons; open arrows, single D2-expressing neurons; filled arrowheads, double CB1/D2 expressing neurons. Blue staining, toluidine blue nuclear counterstaining. Bars, 30 μm in B–E′, 15 μm in F and G.

To dissect the neuronal circuits involved in the effects of THC, we used available conditional mutants lacking CB1 in specific neuronal populations [26,27]. However, these lines present overlapping patterns of deletion, which might limit the interpretation of the results. For instance, GABA-*CB1* ^−/−^ mice and CaMK-*CB1* ^−/−^ mice both lack CB1 expression in striatal medium spiny neurons (MSNs) [27]. As a consequence, possible differences in the effects of THC in these two mouse lines (see below) could be difficult to interpret. Therefore, to obtain a further specific deletion of CB1 in striatal neurons, we crossed *CB1* ^f/f^ mice with a transgenic mouse line where Cre recombinase is expressed under the control of the regulatory sequences of dopamine receptor D1 (D1-Cre) [36,37] to generate D1-*CB1* ^−/−^ mice (Figure 2). Mutant mice were fertile and did not show any obvious phenotypic alteration. In situ hybridization (ISH) analysis of CB1 expression revealed the expected pattern of recombination, with CB1 mRNA absent in the majority of striatal neurons but still expressed in other brain regions (Figure 2A).

Overall, in D1-*CB1* ^−/−^ mice, the great majority of MSNs of caudate putamen do not show CB1 expression, leaving only 27.9% of CB1 expression in the caudate putamen (total CB1-positive cells: D1-*CB1^+/+^*, 13.9 ± 1.3 cells/field versus D1-*CB1* ^−/−^, 3.9 ± 0.2 cells/field; Figure 2A, 2B, and 2B′), which is in agreement with the expected recombination pattern induced by Cre recombinase under the control of D1 regulatory sequences [36]. In the ventral striatum, owing to the very low levels of expression of CB1 mRNA [24,25], it is difficult to quantify the loss of CB1 in mutant mice (Figure 2A). An evaluation based on dark-field images of radioactive ISH for CB1 mRNA (Figure 2C and 2C′) indicate that about 50% of neurons still express CB1 in the mutant mice as compared with wild-type littermates.

Dopamine D1 receptors are expressed not only in striatal neurons, but also in other brain regions. In particular, Cre-mediated recombination under the control of D1 regulatory sequences occurs also in layer VI of the neocortex [36,37]. In this region, CB1 mRNA is present both at high levels in GABAergic interneurons [24] and at low levels in principal glutamatergic neurons, coexpressed with the glutamatergic neuronal marker vesicular glutamate transporter 1 [27] (and unpublished observations). Due to the low levels of CB1 mRNA in these latter neurons and the fact that layer VI has a relatively small dimension, it is difficult to assess the possible deletion of CB1 by an observation of low-magnification pictures (Figure 2A). Therefore, we performed a semi-quantitative counting of the CB1-expressing neurons in layer VI of the neocortex from images captured at high magnification (see Materials and Methods). The results show that the number of high CB1-expressing neurons is not changed in D1-*CB1* ^−/−^ mice (D1-*CB1* ^+/+^, 2.6 ± 0.4 cells/field versus D1-*CB1* ^−/−^ , 2.4 ± 0.4 cells/field, *p* > 0.05, *n* = 5 sections per genotype; Figure 2D and D′), whereas a significant reduction was observed in low CB1-expressing neurons (D1-*CB1* ^+/+^, 17.3 ± 1.4 cells/field versus D1-*CB1* ^−/−^ , 7.7 ± 1.3 cells/field, *p* < 0.01, *n* = 5 sections; Figure 2D and 2D′). In other regions of the brain, including forebrain cortical region (Figure 2A, 2E, and 2E′), forebrain subcortical regions (Figure 2A), midbrain, and hindbrain regions (Figure 2A), no evident alteration was observed in the expression of CB1 in D1-*CB1* ^−/−^ as compared to wild-type D1-*CB1* ^+/+^.

More detailed analysis of CB1 expression in the caudate putamen by double ISH revealed that CB1 is absent in the great majority of non-D2 expressing neurons of mutant mice, indicating the general deletion of the gene in neurons expressing D1 (Figure 2F and 2G). However, in agreement with the expected recombination pattern of D1-Cre mice [36], a certain number of D2-expressing neurons appear to be affected by the Cre-mediated recombination, because the number of double CB1/D2-expressing neurons was decreased in mutant mice. In fact, whereas CB1 is present in 8.3 ± 0.9 cells/field (corresponding to 36.6% ± 2.1% of total D2-expressing neurons) in wild-type D1-*CB1* ^+/+^, this number shows a 5-fold reduction in mutant D1-*CB1* ^−/−^ (to 1.6 ± 0.2 cells/field, corresponding to 7.2% ± 0.6% of total D2-expressing neurons; *p* < 0.0001 [Figure 2F and 2G]). Conversely, not all the cells of caudate putamen that maintained expression of CB1 in D1-*CB1* ^−/−^ belong to the D2-positive population. In fact, in wild-type D1-*CB1* ^+/+^ mice, single CB1-expressing neurons (i.e., non–D2-positive neurons) were counted as 5.7 ± 0.5 cells/field, whereas their presence is not abolished in D1-*CB1* ^−/−^ (2.5 ± 0.1 cells/field). These results indicate that non-D1, non-D2 neurons in the caudate putamen keep their expression of CB1 in the mutant mice. These neurons very likely belong to the population of striatal GABAergic interneurons, which were shown to contain CB1 mRNA [38].

Altogether, these data show that in D1-*CB1* ^−/−^ mice, CB1 expression is strongly reduced in the striatum, with less than 30% of neurons still containing mRNA of the receptor in the caudate putamen and approximately 50% of loss of CB1 expression in the ventral striatum. Of the remaining CB1-positive neurons, one part belongs to the D2-positive subpopulation of MSNs and the other part likely to striatal GABAergic interneurons. Moreover, a significant subgroup of presumably glutamatergic projecting neurons of layer VI of the neocortex shows deletion of CB1 in D1-*CB1* ^−/−^ mice. The recombination pattern of CB1 in D1-*CB1* ^−/−^ mice is similar to the known expression of D1 receptors. However, other cell types might be affected too, such as dopamine receptor D2-expressing MSNs, which are normally considered not to coexpress D1 receptors [39,40]. However, other studies report that the overlap between D1- and D2-expressing neurons in the striatum might be higher than generally believed [41]. It is not the aim of the present study to address this controversy. However, for simplicity, in the following, we will refer to D1-*CB1* ^−/−^ mice as mice with a deletion of CB1 in “dopamine receptor D1-expressing neurons”. This is a given definition and does not imply that Cre-mediated recombination of the *CB1* gene has occurred exclusively in D1-expressing neurons.

### Behavioural Effects of THC in Different CB1 Mouse Mutant Lines

**Figure 3:**
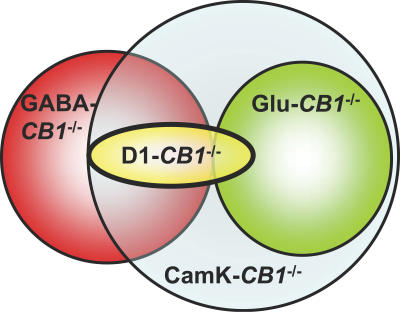
Graphic Representation of the Recombination Patterns of the *CB1* Gene in the Different Conditional Mutant Lines Circles represent “domains“ of recombination. Intersection areas represent overlapping between the different recombination patterns. CaMK*-CB1* ^−/−^ mice (light blue) present the widest recombination of *CB1*, which is partially overlapping with the one of GABA-*CB1* ^−/−^ mice (red-blue overlapping, i.e., principal striatal GABAergic neurons). GABA-*CB1* ^−/−^ mice (red) present a specific deletion of GABAergic neurons, which are specific of this line concerning cortical GABAergic interneurons (non-overlapping red area), but overlap with CaMK-*CB1* ^−/−^ and D1-*CB1* ^−/−^ mice in the striatum. Glu-*CB1* ^−/−^ recombination (green) fully overlap only with CaMK-*CB1* ^−/−^ (glutamatergic cortical neurons). D1*-CB1* ^−/−^ mice (yellow) share with GABA*-CB1* ^−/−^ and CaMK-*CB1* ^−/−^ the deletion in the majority of striatal neurons and with Glu-*CB1* ^−/−^ and CaMK*-CB1* ^−/−^ the deletion in glutamatergic layer VI neocortical neurons. Proportions of overlapping areas are not quantitative and are not representative of cell numbers. Descriptive expression data are collected from previous publications [27,28] and from the present study.

The different mutant mouse lines used in this study, their assigned nomenclature, and a general description of their pattern of expression of CB1 receptor are listed in Table 1. The different mutant lines present specific brain regions and cell types carrying the Cre-mediated deletion of the *CB1* gene [27]. However, overlapping deletions are also present between different lines. In Figure 3, a schematic representation of the patterns of expression of the different lines is shown.

**Table 1:**
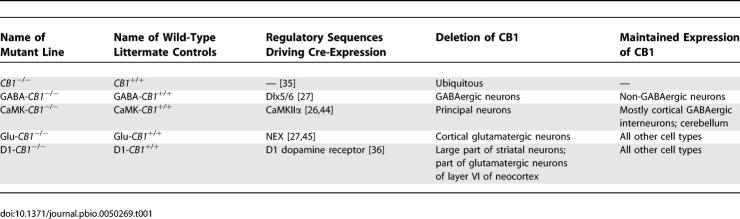
Abbreviations Used to Identify the Different Mutants and Their Wild-Type Littermate Controls

#### Locomotion.

In all mutant lines examined, vehicle-treated wild-type mice showed no significant difference in locomotor activity as compared to the respective mutant littermates (GABA-*CB1* ^+/+^, 3585 ± 206 cm versus GABA-*CB1* ^−/−^ , 3708 ± 195 cm; CaMK-*CB1* ^+/+^, 3339 ± 110 cm versus CaMK-*CB1* ^−/−^ , 3022 ± 182 cm; Glu-*CB1* ^+/+^, 3642 ± 336 cm versus Glu-*CB1* ^−/−^ , 4124 ± 507 cm; D1-*CB1* ^+/+^, 3403 ± 239 cm versus D1-*CB1* ^−/−^ , 3602 ± 328 cm, *p* > 0.05 for all comparisons; *n* = 9–10 for each group), indicating that, in our test conditions, genetic deletion of CB1 did not alter basal locomotor activity of mice.

**Figure 4:**
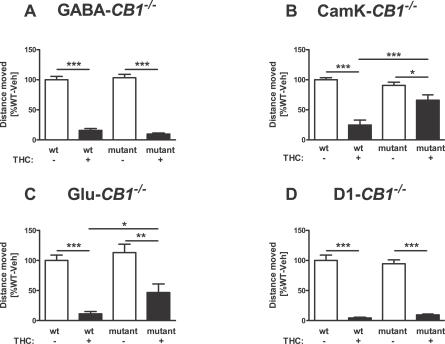
Effect of THC on Locomotor Activity of Different *CB1* Mutant Mice Horizontal locomotor activity was recorded for 15 min, 60 min after the injection of 10 mg/kg THC or vehicle. Values were recorded in cm and normalized to vehicle-treated wild-type animals of each mutant line (100%). *, **, ***; *p* < 0.05, *p* < 0.01, *p* < 0.001, respectively. (two-way ANOVA, followed by Newman-Keuls post-hoc test)*.* Note the normal effect of the drug in GABA-*CB1* ^−/−^ (A), the impaired effect in CaMK-*CB1* ^−/−^ (B), and in Glu-*CB1* ^−/−^ (C), and the normal effect in D1-*CB1* ^−/−^ mice (D). For D1-*CB1* ^−/−^, see text for a slight impairment in the relative effect of THC. See Materials and Methods for number of animals in each group.

Surprisingly, specific deletion of CB1 in GABAergic neurons did not alter the effect of THC on locomotion. Indeed, THC strongly reduced locomotion in both wild-type GABA-*CB1* ^+/+^ and conditional mutant GABA-*CB1* ^−/−^ mice (*p* < 0.0001 Figure 4A), without any significant difference between genotypes (interaction genotype × treatment, F_1,55_ = 1.66, *p* = 0.204; Figure 4A). To reveal more subtle differences between genotypes, we performed further experiments in which a lower dose of THC was used. Also at the dose of 3 mg/kg, no significant difference was observed between genotypes (two-way analysis of variance [ANOVA], Figure S1A and Text S1).

Conversely, in mice lacking CB1 from all principal neurons but still expressing the receptor in a majority of cortical GABAergic interneurons, the hypolocomotor effect of THC was strongly and significantly reduced (Figure 4B). THC decreased locomotion both in CaMK-*CB1* ^+/+^ (*p* < 0.001; Figure 4B) and in CaMK-*CB1* ^−/−^ mice (*p* < 0.05; Figure 4B). However the effect of THC was significantly reduced in the mutant mice as compared to wild-type littermates (interaction genotype × treatment, F_1,36_ = 12.5, *p* = 0.001; Figure 4B)

Together, the results obtained in GABA-*CB1^−/−^* and CaMK-*CB1^−/−^* suggest that the expression of CB1 in GABAergic neurons is not sufficient to exert the effects of THC on locomotor activity, and the results indicate that other neuronal populations must be involved. Interesting candidates for this function are CB1 receptors present in cortical glutamatergic neurons, which project to basal ganglia and thereby likely regulate locomotor activity [42,43]. CaMK-*CB1* ^−/−^ mice are not the ideal model for testing this hypothesis, because in this mouse line, CB1 is absent from all forebrain principal neurons (expressing CaMKIIα) [44], which include cortical glutamatergic neurons and also subcortical projecting neurons in the basal ganglia, thalamus, and hypothalamus [27]. Therefore, we tested the effects of THC in Glu-*CB1* ^−/−^ mice, which lack CB1 expression specifically in cortical glutamatergic neurons, but not in subcortical regions [27,45]. Similarly to its effect on CaMK-*CB1* ^−/−^ mice, THC was able to reduce locomotion both in wild-type Glu-*CB1* ^+/+^ mice (*p* < 0.001; Figure 4C) and Glu-*CB1* ^−/−^ littermates (*p* < 0.01; Figure 4C). However, a significantly lower effect of THC in the mutant mice was observed as compared to wild-type controls (*p* < 0.05, Figure 4C). Further comparison of the relative effects of THC in CaMK-*CB1* ^−/−^ (to 75.4% ± 9% of respective vehicle-treated mice) and Glu-*CB1* ^−/−^ mice (41.2% ± 12% of respective vehicle-treated mice) revealed no significant difference between the two mutant lines (*p* > 0.05, Student's *t*-test), suggesting that CB1 expression in glutamatergic cortical neurons might be a major site mediating the pharmacological effect of THC.

The hypolocomotor effect of THC does not appear to be significantly influenced by expression of CB1 receptors in dopamine receptor D1-containing neurons. The strong effect of THC in wild-type D1-*CB1* ^+/+^ mice was also present in mutant D1-*CB1* ^−/−^ littermates (*p* < 0.001 for both genotypes, Figure 4D), with no significantly different effect in the two genotypes (interaction genotype × treatment F_1,27_ = 0.8, *p* = 0.390). However, although not revealed by two-way ANOVA, a comparison of the relative effects of THC in both genotypes revealed a slight difference between D1-*CB1* ^+/+^ (4.4% ± 1.2%) and D1-*CB1* ^−/−^ (10.1% ± 1.3 % of respective vehicle-treated mice; *p*<0.01, *t*-test), suggesting that THC was slightly less effective in mutant mice.

#### Body temperature.

No significant differences were observed by comparing basal body temperature (before injection of THC or vehicle) of mutant mice with their respective wild-type littermates (GABA-*CB1* ^+/+^, 33.2 ± 0.2 °C versus GABA-*CB1* ^−/−^ , 33.4 ± 0.1°C; CaMK-CB1^+/+^, 35.0 ± 0.1°C versus CaMK-*CB1* ^−/−^ , 35.0 ± 0.1°C; Glu-*CB1* ^+/+^, 33.9 ± 0.1°C versus Glu-*CB1* ^−/−^, 34.0 ± 0.2 °C; D1-*CB1* ^+/+^, 34.1 ± 0.2°C versus D1-*CB1* ^−/−^ , 33.7 ± 0.1°C; *n* = 14–32 for each group; *p* > 0.05 for all comparisons).

**Figure 5:**
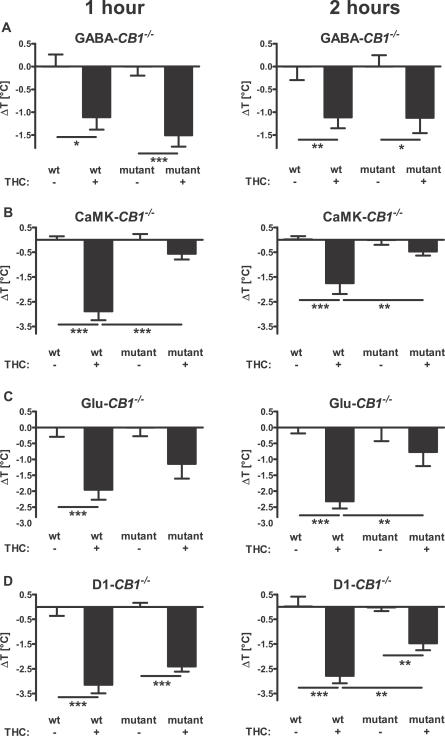
Effect of THC on Body Temperature of Different *CB1* Mutant Mice Body temperature was measured with an infrared thermometer before injection (see text), 1 h (left panels) and 2 h (right panels) after the injection of 10 mg/kg of THC or vehicle. Values represent the difference of the body temperature of each animal with the mean of the respective vehicle-treated animals. *, **, ***; *p* < 0.05, *p* < 0.01, *p* < 0.001, respectively (two-way ANOVA, followed by Newman-Keuls post-hoc test). Note that THC has a normal effect in GABA-*CB1* ^−/−^ (A), but impaired (weaker and/or short lasting) effect in CaMK-*CB1* ^−/−^ (B), in Glu-*CB1* ^−/−^ (C), and in D1-*CB1* ^−/−^ mice (D). See Materials and Methods for number of animals in each group.

Deletion of *CB1* in GABAergic neurons did not alter the effect of THC, with the body temperature of both GABA-*CB1* ^+/+^ and GABA-*CB1* ^−/−^ littermates significantly dropping as compared to respective vehicle-treated controls 1 h and 2 h after injection (*p* < 0.05), without any significant difference in the drug effect between genotypes (1 h, interaction genotype × treatment, F_1,57_ = 0.46, *p* = 0.499; 2 h, interaction genotype × treatment, F_1,56_ = 0.0, *p* = 0.987; Figure 5A). A lower dose of THC was also tested on these mice in a dose-response experiment. Under these conditions as well, the effects of THC were not significantly different between genotypes at both time points after treatment (two-way ANOVA; Figure S1C and S1D, Text S1).

In contrast, the effect of THC was strongly impaired in mice lacking *CB1* in principal neurons. In fact, whereas the drug decreased the body temperature of wild-type CaMK-*CB1^+/+^* mice (1 h, *p* < 0.001 versus vehicle-treated controls; 2 h, *p* < 0.001, Figure 5B), it failed to exert any significant effect on mutant CaMK-*CB1* ^−/−^ littermates (1 h, *p* > 0.05; 2 h, *p* > 0.05 versus vehicle-treated controls), resulting in a significant genotype × treatment interaction at both time points after THC injection (1 h, F_1,36_ = 19.8, *p* < 0.0001; 2 h, F_1,36_ = 6.1, *p* < 0.018; Figure 5B). Similarly to locomotion data, these results indicate that principal neurons, but not GABAergic neurons, mediate the hypothermic effects of THC in mice.

Glutamatergic transmission has been implicated in cannabinoid-induced hypothermia [46]. Therefore, a direct regulation of CB1-expressing cortical glutamatergic afferents might be responsible of the hypothermic effect of THC. Indeed, mutant mice lacking CB1 expression in cortical glutamatergic neurons showed a reduced and shorter-lasting response to the hypothermic effect of 10 mg/kg THC. One and two hours after THC injection, wild-type Glu-*CB1* ^+/+^ mice presented a significant reduction in body temperature as compared to vehicle-injected controls at both time points (*p* < 0.001, Figure 5C), whereas mutant Glu-*CB1* ^−/−^ littermates showed a lower effect, which did not reach statistical significance as compared to respective vehicle-injected controls (*p* > 0.05, Figure 5C). Two-way ANOVA revealed that this situation resulted in a reduction of the drug effect in the mutant mice, which reached statistical significance 2 h after THC injection (interaction genotype × treatment F_1,34_ = 5.4, *p* = 0.026; Figure 5C). Further comparison of the relative effect of THC in CaMK-*CB1* ^−/−^ and Glu-*CB1* ^−/−^ mice revealed no significant difference at either time point after injection (1 h, *p* = 0.3; 2 h, *p* = 0.5), indicating that these two mutant mouse line present a similarly impaired response to hypothermic effects of THC. Altogether, these data strongly indicate that glutamatergic cortical neurons are centrally involved in the hypothermic effects of THC in mice.

Dopamine transmission has been proposed to be involved in the regulation of body temperature [47,48] and to regulate hypothermic effects of THC [49]. Therefore, it is possible that the presence of CB1 in dopamine D1 receptor-expressing neurons contributes to the hypothermic effects of THC. The drug induced a significant decrease of body temperature both in wild-type D1-*CB1* ^+/+^ and mutant D1-*CB1* ^−/−^ littermates at both time points (*p* < 0.01; Figure 5D). However, the effect appeared to be reduced in mutant mice, reaching a significant difference 2 h after drug injection (interaction genotype × treatment F_1,27_ = 5.78, *p* = 0.024; Figure 5D), indicating a lower and shorter lasting effect of THC in the mutant mice.

### Nociception

**Figure 6:**
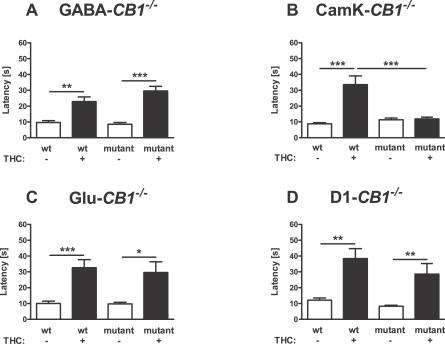
Analgesic Effect of THC in Different *CB1* Mutant Mice Latency to show signs of discomfort on a 55 °C hot plate was measured 75 min after the injection of 10 mg/kg THC or vehicle. A 60-s cut-off time was applied. *, **, ***; *p* < 0.05, *p* < 0.01, *p* < 0.001, respectively (two-way ANOVA, followed by Newman-Keuls post-hoc test). Note that THC has a normal effect in GABA-*CB1* ^−/−^ (A), in Glu-*CB1* ^−/−^ (C) and D1-*CB1* ^−/−^ mice (D), but no effect in CaMK-*CB1* ^−/−^ mice (B). See Materials and Methods for number of animals in each group.

Deletion of CB1 in GABAergic neurons did not alter the analgesic effect of THC. Neither baseline hot-plate escape latencies after vehicle injection (Figure 6A, *p* > 0.05) nor THC-induced analgesia showed any significant difference between wild-type GABA-*CB1* ^+/+^ and mutant GABA-*CB1* ^−/−^ littermates (interaction genotype × treatment F_1,57_ = 1.7, *p* = 0.204; Figure 6A). Identical results were obtained with a lower dose of THC (3 mg/kg), which was also unable to induce significantly different effects between genotypes (two-way ANOVA; Figure S1B and Text S1). Therefore, CB1 receptors that are expressed in GABAergic neurons do not appear to play a significant role in THC-induced analgesia.

Vehicle-treated CaMK-*CB1* ^−/−^ did not show any difference in escape latency as compared to vehicle-treated control wild-type littermates (Figure 6B). Conversely, wild-type CaMK-*CB1^+/+^* mice showed normal sensitivity to the analgesic effect of THC (*p* < 0.001; Figure 6B), whereas the mutant littermates CaMK-*CB1* ^−/−^ did not respond to the injection of the cannabinoid drug (*p* > 0.05; Figure 6B), as shown by a highly significant genotype × treatment interaction (F_1,36_ = 17.9, *p* < 0.001; Figure 6B). These data indicate that principal neurons expressing CB1 receptors play a central role in the analgesic effect of THC.

However, at odds with locomotor and hypothermic effects of THC, cortical glutamatergic neurons do not appear to contribute to the analgesic effect of THC. In fact, the normal effect of the drug observed in wild-type Glu-*CB1* ^+/+^ was present also in mutant Glu-*CB1* ^−/−^ (Figure 6C) with no difference between genotypes (interaction genotype × treatment, F_1,33_ = 0.1, *p* = 0.772), indicating that CB1-postive principal neuronal populations outside of the neocortex are likely involved in these effects of THC.

The deletion of CB1 receptors in dopamine receptor D1-expressing neurons also did not alter the analgesic effects of THC. The drug exerted analgesic effects in both D1-*CB1* ^+/+^ and D1-*CB1* ^−/−^ littermates (*p* < 0.01; Figure 6D), without any significant difference between genotypes (interaction genotype × treatment, F_1,27_ = 0.5, *p* = 0.483), indicating that CB1 expressed in the majority of striatal neurons and in layer VI of neocortex are unlikely to mediate this effect of THC.

### Catalepsy

**Figure 7:**
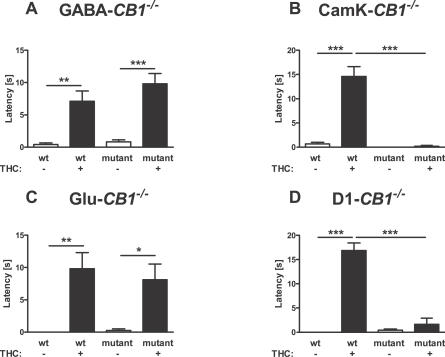
Cataleptic Effect of THC in Different *CB1* Mutant Mice Latency of descent of the forepaws from a horizontal bar was recorded for 20 s, 90 minutes after the injection of 10 mg/kg THC or vehicle. *, **, ***; *p* < 0.05, *p* < 0.01, *p* < 0.001, respectively (two-way ANOVA, followed by Newman-Keuls post-hoc test). The effect is preserved in GABA-*CB1* ^−/−^ (A) and Glu-*CB1* ^−/−^ mice (C), but almost completely abolished in CaMK-*CB1* ^−/−^ (B) and D1-*CB1* ^−/−^ mice (D). See Materials and Methods for number of animals in each group.

THC-induced catalepsy was normally present in both GABA-*CB1* ^+/+^ and GABA-*CB1* ^−/−^ mice (*p* < 0.01 and *p* < 0.001, respectively, Figure 7A), without any difference between genotypes (statistics not shown). A dose-response study confirmed the lack of statistically significant difference between the cataleptic effects of THC in the two groups of mice (two-way ANOVA, Figure S1E and Text S1). Conversely, the cataleptic effect of 10 mg/kg THC could be observed in CaMK-*CB1* ^+/+^ (*p* < 0.001; Figure 7B), but not in CaMK-*CB1* ^−/−^ mice (*p* > 0.05; Figure 7B). Two-way ANOVA revealed a highly significant difference in the effect of THC between the two genotypes (interaction genotype × treatment, F_1,36_ = 44.6, *p* < 0.0001), indicating the dependency of this effect of THC on principal neurons. However, deletion of CB1 from glutamatergic cortical neurons is not sufficient to impair the cataleptic effect of THC. In fact, Figure 7C shows that the drug was able to induce the effect both in Glu-*CB1* ^+/+^ and in Glu-*CB1* ^−/−^ without any significant interaction between treatment and genotype (statistics not shown). Interestingly, 10 mg/kg THC did induce a strong cataleptic effect in D1-*CB1* ^+/+^ mice (*p* < 0.001; Figure 7D), whereas D1-*CB1* ^−/−^ mice were insensitive to this action (*p* > 0.05; interaction genotype × treatment, F_1,31_ = 55.9, *p* < 0.0001; Figure 7D). Thus, GABAergic neurons and cortical glutamatergic neurons do not appear to play a central role in this effect, whereas principal neurons expressing D1 receptors appear to mediate THC-induced catalepsy.

## Discussion

**Figure 8:**
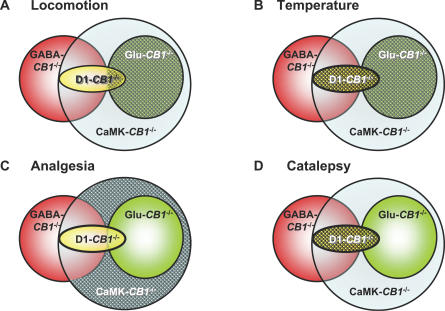
Graphic Representation of Results Obtained with Different *CB1* Mouse Mutant Lines Overlapping of CB1 deletion in the different mutant lines is represented as in Figure 3. Patterned areas represent the likely regions and/or cell types where THC exert its effects on (A) locomotion (principal glutamatergic cortical neurons), (B) body temperature (principal glutamatergic cortical neurons with a possible participation of a portion of striatal MSNs), (C) pain perception (principal neurons, but not striatal MSNs nor glutamatergic cortical neurons), and (D) catalepsy (principal neurons, likely by a simultaneous stimulation of CB1 receptors on both MSNs and glutamatergic cortical neurons). Note that GABAergic interneurons (represented by the nonoverlapping portion of the red circle) never appear to be necessary for any of the observed THC effects.

The present study addressed the neuronal circuits mediating some of the most common effects of cannabinoids in mice, the so-called “tetrad” battery of effects [3,4]. To this aim, we used a combined genetic and pharmacological approach and analysed the effects of THC in previously described CB1 conditional mutant mice [26,27] and in newly generated mice, which lacked CB1 expression in the majority of striatal MSNs and a subset of glutamatergic neurons in layer VI of the neocortex. Taken together (Table 2 and Figure 8), our genetic and pharmacological results show that (1) various pharmacological effects of cannabinoids are mediated by different neuronal circuits, which can be dissected by genetic approaches; (2) GABAergic interneurons do not appear to mediate these effects of THC; (3) cortical glutamatergic neurons mediate a large portion of hypolocomotor effects of cannabinoids; (4) similarly, CB1 expression in cortical glutamatergic neurons plays a prominent role in mediating the hypothermic effects of THC, with a possible partial involvement of a subpopulation of MSNs; (5) the simultaneous activation of CB1 receptors located on striatal neurons and glutamatergic neocortical neurons is likely to be necessary to exert the cataleptic effect of THC; and (6) analgesic effects depend on principal neurons of the central nervous system, but their precise identity is not yet clearly identifiable.

**Table 2:**
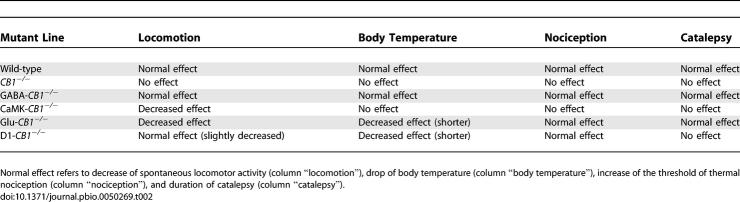
Summary of the Results of THC Treatment in Different CB1 Mutant Lines

Methodological aspects have to be considered in these experiments. CB1 cannabinoid receptors are widely expressed in the central nervous system and, importantly, are present in different neuronal subpopulations [11,17,24,25,50]. This study was undertaken with the aim to dissect important pharmacological effects of THC with respect to their cellular mechanisms and, in particular, to the differential involvement of distinct neuronal subpopulations in these effects. To address this issue, we used conditional mutagenesis, and our results were able to identify several likely sites of action of THC in the brain. However, genetic manipulations are not free of possible confounding issues [51]. Indeed, deletion of a gene by gene targeting (although in a conditional manner) could lead to compensatory mechanisms, which might cause misleading interpretations. Moreover, the use of specific regulatory sequences to drive the expression of Cre recombinase is not devoid of caveats, because the Cre-induced recombination pattern might be different from the expected one, based on the known cell types where the regulatory sequences are supposed to drive Cre expression. For instance, the Nex-Cre mice used to generate Glu-CB1^−/−^ mice, besides showing the expected recombination in cortical glutamatergic neurons, also showed a small degree of recombination in other neurons. The other neurons, however, likely contain very low, if any, expression of CB1 receptors [45]. Nevertheless, conditional mutagenesis is the only tool available to date to dissect with a high degree of precision the role of a given gene in different neuronal populations, and our results, though surprising to a certain extent, fit with other data and concepts present in the literature and, importantly, are confirmed by the parallel analysis of several complementary conditional *CB1* mutant mouse lines.

The persistence of the “tetrad” effects of THC in mice lacking CB1 receptors in GABAergic neurons (Table 2, Figure 8) is surprising. Due to the extremely high levels of CB1 receptors present on cortical GABAergic interneurons, the current hypothesis has been that this neuronal population might mediate most of the effects of exogenously applied or endogenously released (endo)cannabinoids [23]. The lack of THC effects is unlikely to be caused by developmental or compensatory effects of the lack of CB1 in GABAergic neurons in GABA-*CB1* ^−/−^ mice [52] for the following reasons. First, the same effects of THC are abolished in CaMK-*CB1* ^−/−^ and partially reduced in Glu-*CB1* ^−/−^ mice, which still express CB1 in GABAergic interneurons. Second, another important function of CB1 receptors, i.e., the physiological protection against excitotoxic seizures induced by kainic acid, was recently shown to be preserved in GABA-*CB1* ^−/−^ mice and strongly impaired in CaMK-*CB1* ^−/−^ and Glu-*CB1* ^−/−^ mice [27]. Third and importantly, our previous data show that physiological retrograde release of endocannabinoids that act at CB1 receptors expressed in hippocampal GABAergic terminals, mediating short-term forms of synaptic plasticity (depolarization-induced suppression of inhibition, DSI), is abolished in GABA-*CB1* ^−/−^ mice [27], thereby indicating that these mice indeed lack functional expression of CB1 receptors. The overall normal pharmacological effects of THC in GABA-*CB1* ^−/−^ mice, accompanied by the absence of DSI, and the strong reduction of the “tetrad” effects in CaMK-*CB1* ^−/−^ and Glu-*CB1* ^−/−^ mice, accompanied by normal expression of DSI [27], show that these particular endocannabinoid-dependent electrophysiological phenomena are not involved in the “tetrad” pharmacological effects of cannabinoids. These results then suggest that other effects of CB1 activation, such as depression of glutamatergic transmission, might play a more relevant role in this context.

Cannabinoids can control glutamatergic transmission via CB1 receptors in several brain regions [28,53–56]. Recently, using GABA-*CB1* ^−/−^ and CaMK-*CB1* ^−/−^, we could confirm that exogenous application of cannabinoids controls glutamatergic transmission through CB1 receptors present on glutamatergic neurons of the amygdala, the hippocampus, and the neocortex [27,29]. Therefore, it is possible that control of glutamatergic transmission represents the most important neuronal mechanism underlying “classical” effects of cannabinoids in mice. In particular, hypolocomotor and hypothermic effects of THC appear to depend to a large extent on the functional expression of CB1 on cortical glutamatergic neurons, because they are strongly reduced both in CaMK-*CB1* ^−/−^ and in Glu-*CB1* ^−/−^ mice (Table 2). In particular, corticostriatal glutamatergic projection neurons might indeed represent the main site of action of THC to induce hypolocomotor effects (Figure 8A) by reducing the excitatory input onto basal ganglia, as previously shown by electrophysiological recordings [43]. Striatal neurons, being GABAergic principal neurons, are depleted of CB1 expression both in GABA-*CB1* ^−/−^ mice (where THC has a normal effect on locomotion) and in CaMK-*CB1* ^−/−^ mice (where the hypolocomotor effect is strongly reduced). Given the strong impairment of this effect in Glu-*CB1* ^−/−^ mice (which express normal levels of CB1 mRNA in the striatum) [27], the presence of CB1 protein in corticostriatal glutamatergic neurons appears as a plausible candidate to mediate the hypolocomotor effects of THC in mice. To verify this hypothesis, we used mice lacking CB1 expression in the majority of striatal neurons (D1-*CB1* ^−/−^ mice). In these mice, the effect of THC was only very slightly reduced. It is, therefore, possible that a subpopulation of striatal neurons partially contribute to the hypolocomotor effect of THC. However, D1-*CB1* ^−/−^ mice lack CB1 also in a subgroup of pyramidal neurons in the layer VI of neocortex. Given the normal effect of THC in GABA-*CB1* ^−/−^ mice, which lack CB1 in the totality of striatal neurons, but still express normal levels in pyramidal cortical neurons, the most parsimonious interpretation of the data taken as a whole is that the absence of CB1 in layer VI pyramidal neurons of the neocortex likely accounts for the slight reduction of hypolocomotor effect of THC in D1-*CB1* ^−/−^ mice (Figure 8A). However, although striatal projections have been described [57], layer VI pyramidal neocortical neurons project mainly to thalamic regions and their possible function in control of locomotion has been proposed but is still not fully elucidated [58,59]. It is important to note that the hypolocomotor effect of THC in CaMK-*CB1* ^−/−^, Glu-*CB1* ^−/−^, and D1-*CB1* ^−/−^ is not completely abolished in any of the three lines. This might be due to residual expression of CB1 in other brain regions. For instance, cerebellar neurons are not affected by Cre-mediated recombination in either of the lines [27] (Figure 2). Given the importance of this brain region in controlling locomotion, it is very likely that the conserved expression of CB1 in the cerebellum accounts for the portion of maintained effect of THC.

Concerning hypothermic effects, a pharmacological synergy between N-methyl-D-aspartic acid (NMDA) glutamate receptor antagonists and cannabinoids was recently shown in rats [46]. Therefore, inhibition of glutamatergic transmission is a likely mechanism of THC-dependent hypothermia. Moreover, the preoptic anterior hypothalamic nucleus has been proposed to mediate the hypothermic effects of cannabinoids, where they likely regulate glutamatergic transmission [60] (and references inside). Therefore, our data, by showing that hypothermic effects of THC are reduced in CaMK-*CB1* ^−/−^ and Glu-*CB1* ^−/−^ (Table 2 and Figure 8B), are in agreement with the notion that a CB1-dependent reduction of glutamatergic transmission (possibly at the level of cortico-hypothalamic projections) is the main mechanism of THC-induced hypothermic effects. The reduced and shorter lasting effect of THC in D1-*CB1* ^−/−^ mice is more difficult to interpret. On the one hand, the absence of CB1 expression from a subpopulation of glutamatergic neurons of neocortical layer VI might account for the phenotype of these mutant mice. However, a very recent publication suggested that the hypothermic effect of THC and its neuroprotective consequences during cerebral infarction might rely on CB1 expressed in cortical and striatal regions [61]. This suggests that specific striatal neurons might participate in the hypothermic effects of THC.

Hypoalgesic effects of THC, although likely independent from GABAergic interneurons, do not appear to depend on CB1 expressed on cortical glutamatergic neurons nor on striatal neurons, because they are normally present in Glu-*CB1* ^−/−^ and D1-*CB1* ^−/−^ mice, respectively (Table 2, Figure 8C). The exact determination of the neuronal circuitries and the brain regions responsible for each single effect of cannabinoids will require further studies using even more sophisticated experimental approaches, such as viral-induced cell-type–specific deletion of the *CB1* gene. However, the present data already allow some speculation. For analgesic effects, regions such as the periaqueductal grey might play an important role [19,62–64]. However, given the relatively low levels of CB1 expression in these regions of the central nervous system, it is difficult to evaluate the possible lack of expression of CB1 receptors in CaMK-*CB1* ^−/−^ mice, which do not show THC-induced hypoalgesia. Nevertheless, our data clearly show that GABAergic neurons are not implicated in this effect. Moreover, it is possible that hypoalgesic effects of cannabinoids depend on the expression of CB1 at spinal sites and/or even in peripheral neurons [19,64,65]. Indeed, recent data clearly showed that conditional deletion of the *CB1* gene in peripheral neurons strongly reduces analgesic effects of cannabinoid drugs [65]. In addition, interaction with noradrenergic neurons in the spinal cord might be a mechanism of THC-induced hypoalgesia [66]. Therefore, given the wide expression of CaMKIIα at several sites (likely including also spinal and peripheral neurons), the lack of hypoalgesic effect of THC in CaMK-*CB1* ^−/−^ cannot, at the moment, be ascribed to a precise location in the complex pathways mediating pain perception.

Cataleptic effects of THC are impaired both in CaMK-*CB1* ^−/−^ and in D1-*CB1* ^−/−^ mice (Table 2, Figure 8D). The basal ganglia are involved in the adjustment and fine tuning of voluntary movements through two major pathways: the D1-type dopamine receptor-containing direct and the D2-type dopamine receptor-containing indirect pathways [42]. Interestingly, catalepsy can be observed by pharmacological manipulation of both pathways, and its exact mechanisms are not yet understood in detail [67–69]. Moreover, catalepsy is one of the hallmark effects of treatments, such as 6-OHDA and reserpine, that are able to induce parkinsonian symptoms in animals [56]. In CaMK-*CB1* ^−/−^ mice, CB1 receptors are lost in all projecting neurons of the brain, thus making it difficult to define the exact site of action of THC to induce catalepsy. However, noradrenergic and serotonergic pathways (involving 5HT1a receptors) were proposed to play a role in THC-induced catalepsy [70,71]. Noradrenergic and serotonergic neurons likely express very low, but significant levels of CB1 [72,73]. As these neurons are principal projecting neurons, it is possible that they lack CB1 expression in CaMK-*CB1* ^−/−^. Thus, lack of THC-induced control of serotonergic and/or noradrenergic transmission might participate in the phenotype of CaMK-*CB1* ^−/−^. In the D1-*CB1* ^−/−^ mice, on the other hand, most incoming striatal afferents from cortex and thalamus still express CB1 normally (apart from some neurons of neocortical layer VI). However, MSNs of the direct pathway (traditionally considered as D1-positive) [57] lack CB1 receptors, together with a certain amount of putative neurons belonging to the D2-positive subpopulation (believed to constitute the indirect pathway). Therefore, cannabinoid treatment cannot affect GABA release at the output sites in the globus pallidus and the substantia nigra. Yet, GABA-*CB1* ^−/−^ mice, lacking CB1 from all GABAergic neurons (including all striatal principal neurons) express catalepsy normally after THC treatment. Cannabinoid-induced catalepsy, therefore, is produced most probably by a simultaneous disturbance at more than one site in the basal ganglia motor pathway. In this regard, the lack of CB1 from a subgroup of pyramidal neurons in the neocortical layer VI of D1-*CB1* ^−/−^ mice might be particularly interesting. Indeed, it is possible to speculate that the simultaneous action of THC at CB1 receptors expressed both in layer VI cortical neurons and in D1-expressing MSNs is necessary to exert the typical cataleptic effect of the drug. In this frame, the presence of CB1 in cortical glutamatergic neurons of GABA-*CB1* ^−/−^ mice would be sufficient to exert the normal cataleptic effect of THC. Interestingly, MSNs belonging to the direct and indirect pathways were recently shown to be differentially regulated by endocannabinoids, and this phenomenon might have a particular importance in the pathophysiology of Parkinson disease [56]. Therefore, it will be very interesting to explore the phenotype of D1-*CB1* ^−/−^ in models of this disease, in order to start dissecting the loci where cannabinoid-based therapy might be useful in the treatment of this important neurological disorder [15,18,42].

The “tetrad” battery of effects does not represent the myriad pharmacological functions of cannabinoids, ranging from effects on learning [74,75], stress responses [12,75], neuroendocrine and energy balance [12], reward [76], and many others [15]. However, the fact that it is now possible to dissect each of these effects will pave the way to a novel concept of cannabinoid pharmacology and to new insights into its mechanisms. This might also include the future possibility for therapeutic targeting of specific cannabinoid effects exerted in distinct neuronal subpopulations. It is becoming more and more evident that CB1 receptors physically interact with specific proteins in different neuronal populations and that these interactions are able to modify their pharmacological profile [77–80]. Therefore, it will be possible in the future to identify cannabinoid ligands that are able to interact with CB1 receptors specifically coupled or uncoupled with other proteins and, thereby, expressed in different populations. The use of these drugs and the precise determination of the sites of action of cannabinoids for different effects might provide the opportunity to exploit better the therapeutic potentials of cannabinoids, avoiding possible undesirable side effects.

## Materials and Methods

### Animals.

Male mice, aged 2–5 mo, were used in all experiments, maintained in standard conditions with food and water ad libitum. All experimental procedures were approved by the Committee on Animal Health and Care of the local government. Conditional CB1 mutant mice were obtained by using the Cre/loxP system [31]. The respective Cre-expressing mouse line was crossed with *CB1^f/f^* mice [26], using a three-step breeding protocol. CaMK-*CB1^−/−^*, Glu-*CB1^−/−^*, and GABA-*CB1^−/−^* mice were obtained as described [26,27]. Genotyping was performed by PCR as described for CaMK-*CB1^−/−^* and for *CB1^f/f^* [26]. For the GABA-*CB1^−/−^* line, genotyping for the Cre transgene was performed by PCR using the following primers: forward 5′- GAT CGC TGC CAG GAT ATA CG; reverse: 5′ - CAT CGC CAT CTT CCA GCA G, whereas genotyping for the *CB1^f/f^* locus was performed as described [26]. *CB1^+/+^* and *CB1^−/−^* mice were generated and genotyped as described [35]. All lines were in a mixed genetic background, with a predominant C57BL/6NCrl contribution. All animals used in single experiments were littermates. Experimenters were always blind to genotype and treatment.

The abbreviations used to identify the different mutants and their wild-type littermates are summarized in Table 1.

### Generation of D1-*CB1^−/−^* mice.

To generate the D1-*CB1^−/−^* line (Figure 1), *CB1^f/f^* mice [26] were crossed with dopamine receptor D1-Cre line [36,37], in which the Cre recombinase was placed under the control of the dopamine receptor D1A gene *(Drd1a)* regulatory sequences using transgenesis with modified bacterial artificial chromosomes. The pattern of Cre expression recapitulated the expression pattern of the endogenous *Drd1a* [36,37]. Genotyping for the Cre transgene was performed by PCR using the following primers: forward 5′- GAT CGC TGC CAG GAT ATA CG; reverse: 5′ - CAT CGC CAT CTT CCA GCA G, whereas genotyping for the *CB1^f/f^* locus was performed as described [26].

### Drugs.

Δ^9^-tetrahydrocannabinol (THC, Sigma-Aldrich; http://www.sigmaaldrich.com) was purchased as a 10 mg/ml (w/v) solution in 100% ethanol. This solution was concentrated to 100 mg/ml using a SpeedVac and, immediately before injection, mixed with Tween 80, and then diluted with 0.9% saline and shaken for 10 min at 37 °C. Vehicle control contained all ingredients (1 drop / 3 ml of Tween 80 and ethanol diluted 1:40 with saline) except Δ^9^-THC. Drug/vehicle was administered intraperitoneally with an injection volume of 10 ml/kg body weight. For behavioural tests, mice of each genotype received different doses of THC or vehicle, as previously described [4,81].

### Behavioural tests.

To determine the effects of THC the following number of animals were used in the experiments (Veh indicates vehicle): GABA-*CB1^+/+^*-Veh, 10; GABA-*CB1^+/+^*-THC, 19; GABA-*CB1^−/−^*-Veh, 10; GABA-*CB1^−/−^*-THC, 20; CaMK-*CB1^+/+^*-Veh, 9; CaMK-*CB1^+/+^*-THC, 10; CaMK-*CB1^−/−^*-Veh, 10; CaMK-*CB1^−/−^*-THC, 11; Glu-*CB1^+/+^*-Veh, 9; Glu-*CB1^+/+^*-THC, 10; Glu-*CB1^−/−^*-Veh, 8; Glu-*CB1^−/−^*-THC, 9; D1-*CB1^+/+^*-Veh, 9; D1-*CB1^+/+^*-THC, 9; D1-*CB1^−/−^*-Veh, 10; D1-*CB1^−/−^*-THC, 11.

Animals were placed in a reversed dark/light cycle (light off 8 a.m., light on 8 p.m.) for at least 15 d before the experiments. All behavioural tests were performed in the dark phase (between 10 a.m. and 3 p.m.) under dim red light illumination in the following order. Basal body temperature was measured in all animals before injection. One hour after the injection of THC or vehicle, temperature was again measured and animals were placed in the open field to assess locomotion for 15 min. Immediately afterwards, the mice were placed on the hot-plate test and analgesia was measured for maximum of 60 s (see below). Mice returned to their home cage for about 14 min and then catalepsy was tested. After returning to home cage for another 13–14 min, the temperature was measured again (2 h after drug injection).

### Locomotor activity.

Locomotion was measured 60 min after injection of THC or vehicle by an automated open field system (box size 32 × 32 cm; illumination of 0–10 lux, MOTION, TSE GmbH; http://www.tse-systems.com). Animals were individually tested for 15 min. The cumulative horizontal distance the animals moved within the box was recorded.

### Analgesia.

THC-induced analgesia was measured using a hot plate analgesia meter (type 12801, Bachofer Laboratoriumsgeräte, Reutlingen, Germany) 75 min after injection of the drug or the vehicle. The plate was heated to 55 ± 0.5 °C and the time until mice showed the first sign of discomfort (licking or flinching of the paws or jumping on the plate, here defined as escape latency) was recorded. A cut-off time of 60 s was set to prevent tissue damage.

### Body temperature.

Body temperature was measured immediately prior, as well as 60 and 120 min after injection of drug or vehicle using a C-1600 infrared thermometer (Linear Laboratories; http://www.linearlabs.com), which was placed between the forepaws at a distance of approximately 3 cm from the abdomen.

### Catalepsy.

THC-induced catalepsy was measured by the bar catalepsy test 90 min after drug or vehicle injection. The forepaws of mice were placed on a 1-cm-diameter bar fixed horizontally at 3.5 cm from the bench surface. The descent latency was recorded for an observation period of 20 s.

### In situ hybridization.

Single and double ISH was carried out as previously described [24,82] using radioactive and nonradioactive CB1- and radioactive dopamine receptor D2-specific riboprobes [24,82], respectively. Counting of cells expressing CB1 in the VI layer of the neocortex of D1-*CB1^−/−^* and control D1-*CB1^+/+^* littermates was carried out on single radioactive ISH experiments. Random regions corresponding to the VI layer of neocortex (sensory-motor part, right above the corpus callosum) were captured at 40× magnification. Numbers were randomly assigned to the images, and an observer (blind of the genotype of each single image) counted high CB1-expressing neurons (corresponding to GABAergic interneurons and defined as in [24]) and low CB1-expressing neurons (belonging to pyramidal glutamatergic cortical neurons) [27]. Numbers of low and high CB1-expressing neurons per analyzed field were calculated. For CB1/D2 coexpressing neurons in the striatum, a similar approach was used in double ISH slides. Numbers of single CB1-, single D2- and double-expressing neurons were calculated.

### Data analysis.

Data are presented as mean ± standard error of the mean (SEM) of individual data points. Vehicle-mediated effects were compared between genotypes in absolute values. For open field, data are expressed as percentages of vehicle-treated wild-type animals (absolute data of vehicle-injected mice are reported in the text). Body temperature data are expressed as differences from vehicle-treated animals of the same genotype (defined as Δ*T* in the figures), with absolute values of vehicle-treated mice reported in the text. Data were analysed using two-way ANOVA, using genotype and treatment as variables, and Newman-Keuls post-hoc test. In some cases, to appreciate differential effects of THC between different strains, percentage “relative effects” of THC were calculated for each genotype and analyzed with Student's *t*-test. Graphs and statistics were generated by GraphPad Prism 4.03 (GraphPad Software; http://www.graphpad.com) and Statistica 5.0 (StatSoft; http://www.statsoft.com), respectively.

## Supporting Information

Figure S1 Dose-Response of THC Effects on GABA-*CB1^+/+^* and GABA-*CB1^−/−^* Mice (49 KB PDF)

Text S1 Materials and Methods Describing Figure S1
(30 KB DOC)

## Abbreviations

6-OHDA: 6-hydroxy-dopamine
ANOVA: analysis of variance
CaMKIIα: Ca^2+^/calmodulin-dependent kinase IIα
CB1: cannabinoid receptor type 1
D1: dopamine receptor type 1
D2: dopamine receptor type 2
DSI: depolarization-induced suppression of inhibition
GABA: γ aminobutyric acid
ISH: in situ hybridization
MSN: striatal medium spiny neuron
THC: Δ^9^-tetrahydrocannabinol
