# Supplementary material for: Genetic Dissection of Behavioural and Autonomic Effects of Δ9-Tetrahydrocannabinol in Mice
Source: PLoS Biol. 2007 Oct 9;5(10):e269. doi: 10.1371/journal.pbio.0050269 (PMC2001214; doi:10.1371/journal.pbio.0050269)
Supplement: Supplementary file 1 [file pbio.0050269.sg001.pdf]

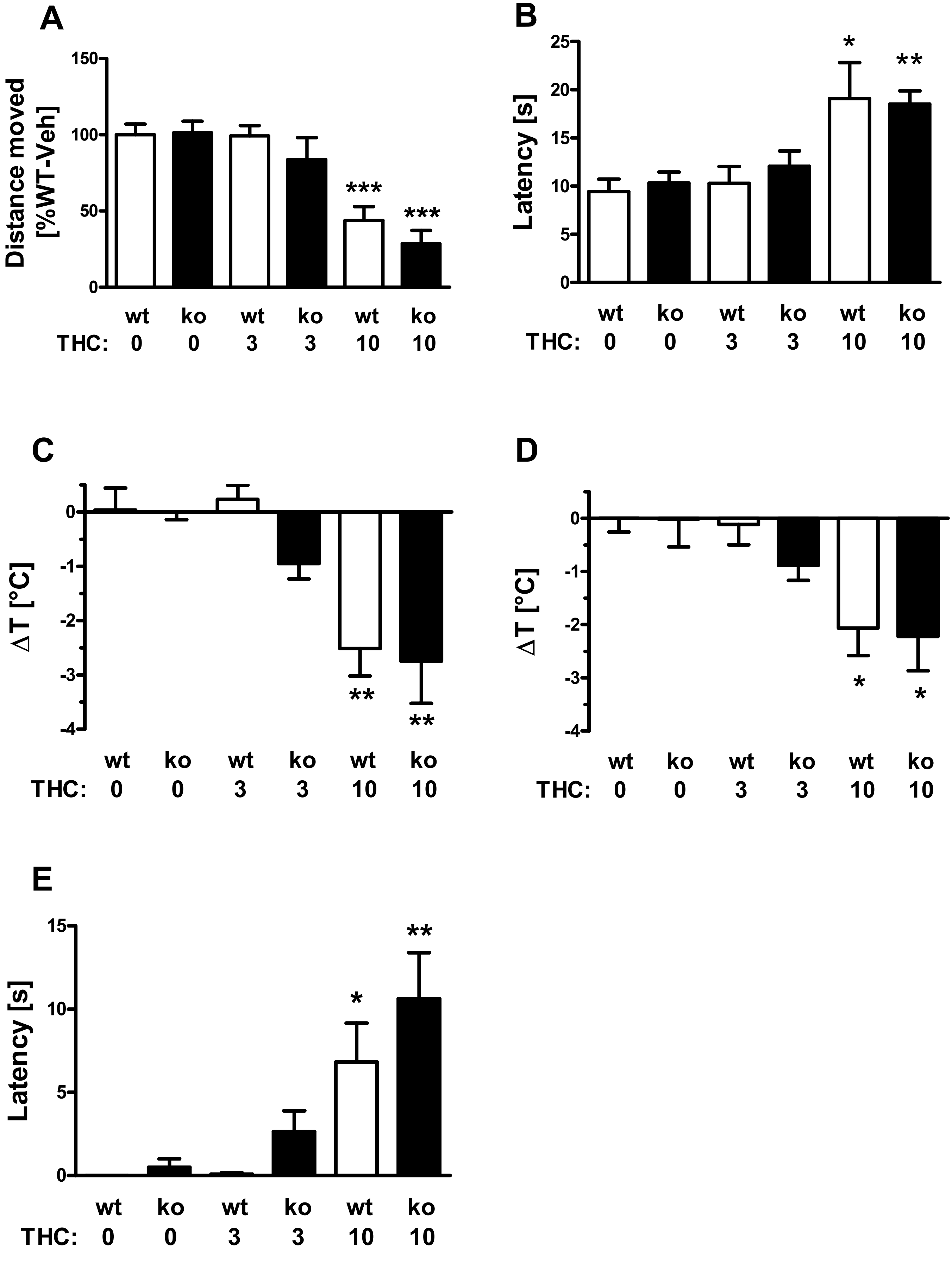

Monory et al. Supplementary Figure 1
