# Supplementary material for: Genetic Dissection of Behavioural and Autonomic Effects of Δ9-Tetrahydrocannabinol in Mice
Source: PLoS Biol. 2007 Oct 9;5(10):e269. doi: 10.1371/journal.pbio.0050269 (PMC2001214; doi:10.1371/journal.pbio.0050269)
Supplement: Supplementary file 2 [file pbio.0050269.sd001.doc]

**Text S1**

**Dose-response of THC effects in** **GABA-*CB1*^+/+^ and GABA-*CB1*^-/-^ mice.**

**Materials and Methods**

***Drugs.*** Δ^9^-tetrahydrocannabinol was from THC Pharm (Frankfurt, Germany). Following manufacturer’s instructions, the glass syringe containing the wax was warmed up with heat gun, the liquid compound harvested in an Eppendorf-tube, measured and dissolved in ethanol in a concentration of 100 mg/ml. This solution was, immediately before injection, mixed with Tween 80, and then diluted with 0.9% saline and shaken for 10 min at 37°C. Vehicle control contained all ingredients (1 drop / 3 ml of Tween 80 and ethanol diluted 1:40 with saline) except THC. Drug/vehicle was administered intraperitoneally with an injection volume of 10 ml/kg body weight. For behavioural tests, mice of each genotype received 3 mg/kg of THC / 10 mg/kg of THC / vehicle, respectively.

***Behavioural tests.***

All behavioural tests were performed in the dark phase of the circadian rhythm. The experimental groups consisted of the following number of animals: *CB1*-GABA^+/+^-Veh, 7; *CB1*-GABA^+/+^-THC (3 mg/kg), 12; *CB1*-GABA^+/+^-THC (10 mg/kg), 11; *CB1*-GABA^-/-^-Veh, 6; *CB1*-GABA^-/-^-THC (3 mg/kg), 8; *CB1*-GABA^-/-^-THC (10 mg/kg), 8.

**Locomotor activity.** Locomotion was measured 60 minutes after injection of THC or vehicle in a 40 x 40 cm box for 15 minutes. The open field arena was recorded with a Sony HAD XC 129 camera. Video data were fed into a PC and directly analysed by video analysis software (SMART, Panlab, Madrid, Spain) and simultaneously recorded on a Panasonic LM-PR 120 ME 10 DVD-recorder for possible re-analysis / archiving purposes. The cumulative horizontal distance the animals moved within the box was calculated.

**Analgesia.** THC-induced analgesia was measured using a hot plate analgesia meter (Linton, Norfolk, UK) 75 min after injection of the drug or the vehicle. The plate was heated to 55 ± 0.5 C and the time until mice showed the first sign of discomfort (licking or flinching of the paws or jumping on the plate, here defined as escape latency) was recorded. A cut-off time of 60 sec was set to prevent tissue damage.

**Body temperature.** Body temperature was measured immediately prior, as well as 60 and 120 min after injection of drug or vehicle using an C-1600 infrared thermometer (Linear laboratories, Fremont, CA, USA), which was placed between the forepaws at a distance of approximately 3 cm from the abdomen.

**Catalepsy.** THC-induced catalepsy was measured by the bar catalepsy test 90 minutes after drug or vehicle injection. The forepaws of mice were placed on a 1-cm diameter bar fixed horizontally at 3.5 cm from the bench surface. The descent latency was recorded for an observation period of 20 seconds.

**Supplementary Figure Legend**

**Figure S1.** Dose-response of THC effects on GABA-*CB1*^+/+^ and GABA-*CB1*^-/-^ mice. **A)** Locomotion. No significant difference was observed between genotypes (2-way ANOVA; F_1,47_ = 1.9, p > 0.05). **B)** Hypoalgesia. No significant difference was observed between genotypes (F_1,47_ = 1.4, p > 0.05 **C, D)** Body temperature. No significant difference was observed between genotypes (2-way ANOVA; 1 hour: F_1,47_ = 0.7, p > 0.05; 2 hours: F_1,47_ = 0.8, p > 0.05). **E)** Catalepsy. No significant difference was observed between genotypes (2-way ANOVA; F_1,47_ = 3.0, p > 0.05). *, **, ***, p < 0.05, p < 0.01, p < 0.001, respectively, as compared to the respective vehicle-treated groups (2-way ANOVA, followed by Newman-Keuls post-hoc test). These data indicate that the effects of THC are not dependent on the presence of CB1 receptors on GABAergic neurons.
